# Synoviolin alleviates GSDMD‐mediated periodontitis by suppressing its stability

**DOI:** 10.1002/iid3.880

**Published:** 2023-07-12

**Authors:** Ying Pang, Lili Liu, Shuainan Wu, Jianqi Wang, Lu Liu

**Affiliations:** ^1^ Dental Clinic, Cangzhou Central Hospital Cangzhou Hebei People's Republic of China

**Keywords:** GSDMD, inflammasome, periodontitis, Synoviolin

## Abstract

**Introduction:**

Inflammasome and pyroptosis play important roles in periodontitis. Gasdermin D (GSDMD), a key factor in pyroptosis, is cleaved by caspase‐1 and regulated by ubiquitination. Synoviolin is a ubiquitin E3 ligase that interacts with GSDMD. In this study, the effects of Synoviolin on inflammasome activation and periodontitis were explored.

**Methods:**

The expression of IL‐1β, GSDMD, and Synoviolin in peripheral blood mononuclear cells from patients with periodontitis was determined. The interaction between GSDMD and Synoviolin was studied. The cytokine level in gingival tissues and the distance from the cementoenamel junction to the alveolar bone crest were measured in mice with Synoviolin deficiency in myeloid cells.

**Results:**

We reported that elevated mRNA and protein levels of IL‐1β and GSDMD, decreased levels of Synoviolin mRNA and protein, and decreased ubiquitination of GSDMD were associated with periodontitis. Synoviolin interacted with GSDMD. Synoviolin‐deficient bone marrow‐derived macrophages had increased IL‐1β and IL‐18 secretion after ATP stimulation. Mice with Synoviolin deficiency in myeloid cells had more severe periodontitis and upregulated IL‐1β and IL‐18.

**Conclusions:**

Therefore, we conclude that Synoviolin suppresses inflammasome activation and periodontitis by regulating GSDMD stability.

## Graphical abstract

1. Synoviolin interacted with GSDMD. 2. Synoviolin‐deficient BMDMs had increased IL‐1β and IL‐18 in cell supernatant after ATP stimulation.

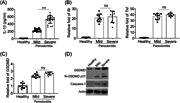

## 1 INTRODUCTION

Periodontitis is an inflammatory disease destroying the tooth‐supporting apparatus.^1^ Inflammation plays a critical role in periodontitis. The tissue destruction in periodontitis starts with the intensive inflammatory response to periodontal pathogenic bacteria. Periodontitis is associated with Gram‐negative anaerobic bacteria, including *Porphyromonas gingivalis*(Pg), *Aggregatibacter actinomycetemcomitans*, *Treponema denticola*, *Fusobacterium nucleatum*, and *Tannerella forsythia*.^2^ The antigen components, enzymes, toxins, and metabolites of these periodontal pathogens could induce local immune and inflammatory reactions, resulting in periodontal tissue damage. In periodontitis, various proinflammatory cytokines such as IL‐1β, IL‐6, and tumor necrosis factor‐α (TNF‐α) are produced. Elevated levels of IL‐1β, IL‐6, and TNF‐α have been detected in inflamed gingival tissues of patients with periodontitis, which contributes to tissue destruction and the progress of periodontitis.^3^, ^4^, ^5^ IL‐1β is an important cytokine that induces the expression of other mediators and matrix metalloproteinases and leads to the destruction of connective tissues and alveolar bones and bone absorption.^6^, ^7^ IL‐6 is associated with inflammatory cell migration and is implicated in bone hemostasis by regulating osteoclast differentiation and bone resorption,^8^ and osteoclast is important in bone absorption.^9^ TNF‐α participates in bone metabolism and exacerbates bone resorption and damages the oral mucosal barrier.^7^ IL‐18 is an interferon‐gamma‐inducing factor and an activator of Th1 and NK cells. During Pg infection, IL‐18 is overexpressed, which leads to inflammatory bone loss and activation of antigen‐specific lymphocytes.^10^ Cytokine production by peripheral blood mononuclear cells (PBMCs) can reflect the activity of immuno‐inflammatory diseases in specific organs and tissues.^11^ PBMC cells from periodontitis subjects released higher levels of TNF‐a and IL‐6 than those from healthy subjects after *Escherichia coli* lipopolysaccharides stimulation.^12^

IL‐1β and IL‐18 are expressed as inactive precursors termed pro‑IL‑1β and pro‐IL‐18.^13^, ^14^ The conversion of pro‑IL‑1β and pro‐IL‐18 into the activated form IL‑1β and IL‐18 is mediated by a multiprotein complex termed the inflammasome, which regulates several diseases including periodontitis.^15^ The inflammasome consists of a pattern recognition receptor (PRR), an adaptor protein apoptosis‐associated speck‐like protein containing a CARD (ASC), and an active form of caspase‐1.^16^ Once the PRR binds to its ligand, the ASC is recruited and it converts procaspase‐1 to active caspase‐1, which subsequently cleaves pro‐IL‐1β and pro‐IL‐18 into their biologically active forms. Caspase‐1 also cleaves gasdermin D (GSDMD), and the lytic N‐terminal domain (N‐GSDMD) of GSDMD is released and oligomerizes to form pores in the cell membrane, allowing the release of mature IL‐1β and IL‐18 and inducing pryoptosis.^17^ Pyroptosis is a process of cellular self‐destruction mediated by caspases. Caspase‐1 cleaves GSDMD, resulting in cell membrane perforation through the release of the GSDMD N‐terminal fragment. The knowledge of pyroptosis in the pathogenesis of periodontitis is evolving. Studies conducting bioinformatics analyses have identified pyroptosis‐related genes, revealing the crucial role of pyroptosis in modifications of the immune microenvironment in periodontitis.^18^, ^19^, ^20^ Dysregulation of GSDMD has been implicated in several diseases. For example, a greatly enhanced level of GSDMD protein has been found in the CNS of EAE mice.^21^ The expression of GSDMD was increased in patients with Alzheimer's disease (AD), which could be used as a biomarker of AD.^22^

Studies have revealed that nucleotide‐binding and oligomerization domain‐like receptor (NLR) pyrin domain‐containing 3 (NLRP3) and absent in melanoma 2 (AIM2) inflammasome are involved in periodontal disease pathogenesis.^23^, ^24^, ^25^, ^26^ The NLRP3 inflammasome consists of NLRP3, ASC, and caspase‐1 and could be activated by cell stress, bacteria, and viruses.^27^, ^28^, ^29^ AIM2 is a nonNLR inflammasome that senses double‐stranded DNA from various sources, including bacteria, viruses, or host cells.^30^ It has been described that Pg activated the NLRP3 inflammasome and promoted alveolar bone loss in wild‐type (WT) mice. In contrast, these phenomena disappeared in NLRP3‐deficient mice.^31^

Synoviolin, also known as Hrd1, is one of the E3 ubiquitin ligases involved in endoplasmic reticulum‐associated degradation.^32^ Synoviolin is known to target multiple substrates. For example, Synoviolin promoted the degradation of proapoptotic factor IRE1.^33^ Synoviolin ubiquitinated B lymphocyte‐induced maturation protein 1 and promoted its degradation.^34^ Synoviolin ubiquitinated and inactivated Usp15 and promoted inflammation during bacterial infection.^35^ A recent study by Shi et al. described that Synoviolin directly interacted with GSDMD, mediated K27‐linked polyubiquitination of GSDMD, and promoted GSDMD‐induced pyroptosis in HEK293T cells.^36^ Until now, the precise roles of Synoviolin in periodontitis have never been described. Since GSDMD and pyroptosis are involved in periodontitis, we hypothesized that Synoviolin might regulate GSDMD and pyroptosis in periodontitis by ubiquitinating GDDMD and promoting its degradation. In this study, we explored the functions of Synoviolin in periodontitis.

## 2 MATERIALS AND METHODS

### 2.1 Patients' samples

Serum and PBMCs from healthy donors and patients with mild and severe periodontitis were collected at Cangzhou Central Hospital. The healthy donors defined as having no periodontitis or other systemic inflammation were detected (male = 10, female = 10). The specific classification of periodontal disease has been described previously^37^ and the following are the criteria for defining periodontitis: (1) Interdental clinical attachment loss (CAL) is detectable at ≥2 nonadjacent teeth; (2) Buccal or oral CAL ≥ 3 mm with pocketing >3 mm is detectable at ≥2 teeth. Periodontitis (mild) patients have probing depths ≤5 mm, CAL ≤ 4 mm, horizontal bone loss, and require nonsurgical and surgical treatment. No posttreatment tooth loss is expected, indicating the case has a good prognosis going into maintenance (male = 9, female = 11). Periodontitis (severe) patients have probing depths ≥5 mm, CAL ≥ 4 mm, and have vertical bone loss and/or furcation involvement. This condition requires surgery and possibly regenerative treatments. There is potential for tooth loss. The complexity of implant and/or restorative treatment is increased. The patient requires multispecialty treatment. The overall case has a fair prognosis going into maintenance (male = 10, female = 10). The mean age of participants is 42.7. Patients with the following criteria were excluded: (1) with Systemic diseases such as diabetes, blood disorders, and diseases of the immune system; (2) history of antibiotic and periodontal therapy within the 4 months prior to study or allergy to the macrolide group of antibiotics; (3) smoking, pregnant, or lactating females; (4) patients treated with drugs such as antiacid, warfarin, and cyclosporine and alcohol use. All participants were provided with informed consent. The study was approved by the Ethics Committee of Cangzhou Central Hospital (#2020/05/d63).

### 2.2 Animals

Eight‐week‐old female C57BL/6 mice were purchased from the animal model research center of Nanjing University. Synoviolin floxed mice were purchased from Shanghai Model Organism. To generate myeloid‐specific knockout (KO) mice, Synoviolin floxed mice were crossed with B6‐ Lyz2‐Cre mice (Shanghai Model Organism). The genotypes of mice were determined by PCR analysis. All animal works were approved by the Institutional Animal Care and Use Committee in Cangzhou Central Hospital (#JCYJ.2020.03.c1).

### 2.3 Cells

Bone marrow‐derived macrophages (BMDMs) were generated following the standard protocol as described previously.^38^

### 2.4 Mice model of periodontitis (PD)

Colony forming units of, 2 × 10^9^, Pg strain 381 were employed. The Pg strain 381 was cultured anaerobically and grown in a gifu anaerobic medium that contained 5 mg/mL of hemin and 5 μg/mL of vitamin K at 37°C. Pg was plated in 100 μL of phosphate‐buffered saline with 2% carboxymethylcellulose. Mice were anesthetized by intraperitoneal injection of 10% chloralhydrate (4 mL/kg). Then the subgingival of the first and second molars were ligated with silk sutures (3/0) presoaked in Pg solution, with a continuous “∞” approach on either side of the maxilla bone. The ligation was checked twice weekly and was replaced if it had been displaced or loosened. Four weeks later, the ligatures were removed. The right half maxillaries of the mice model were harvested for micro‐CT analysis.

### 2.5 Micro‐CT analysis

Specimens were scanned using the MicroCT system (µCT100, Seanco Medical). The distance from the cementoenamel junction (CEJ) to the alveolar bone crest (ABC) was measured to obtain the height of alveolar bone loss. The mouse maxillary alveolar bone was harvested and fixed in 10% neutral buffered formalin for 24 h. The harvested tissue was transferred into 70% ethanol for scanning at a resolution of 10.5 µm and 55 kVp. All scans were reoriented with DataViewer in the same position to evaluate the bone loss. The CEJ‐ABC distance was measured at a 52.5 µm interval.

### 2.6 Real‐time PCR

The total RNA of PBMCs and periodontal tissues was extracted using GeneJET RNA Purification Kit (Thermo Fisher). RevertAid First Strand cDNA Synthesis Kit was used to synthesize cDNA. The real‐time PCR was performed using SYBR™ Green PCR Master Mix and 7500 Fast Real‐Time PCR System (Thermo Fisher). The primers used in this study included: Human Il1β sense 5′‐CCACAGACCTTCCAGGAGAATG−3′ Human Il1β antisense 5′‐ GTGCAGTTCAGTGATCGTACAGG−3′; Human Tnf sense 5′‐CTCTTCTGCCTGCTGCACT TTG−3′ Human Tnf antisense 5′‐ATGGGCTACAGGCTTGTCACTC−3′; Human GSDMD sense 5′‐ATGAGGTGCCTCCACAACTTCC−3′ Human GSDMD antisense 5′‐CCAGTTCC TTGGAGATGGTCTC‐3′; Human Synoviolin (Synv1) sense 5′‐CCAACATCTCCTGGCTCTTTCAC‐3′ Human Synoviolin antisense 5′‐GTCAGGATGCTGTGATAGGCGT‐3′; Mouse Synoviolin sense 5′‐ CCAACATCTCCTGGCTCTTCCA‐3′ Mouse Synoviolin antisense 5′‐CAGGATGCTGTGATAA GCGTGG‐3′; Mouse Il1β sense 5′‐TGGACCTTCCAGGATGAGGACA‐3′ Mouse Il1β antisense 5′‐ GTTCATCTCGGAGCCTGTAGTG‐3′; Mouse Il18 sense 5′‐GACAGCCTGTGT TCGAGGATATG‐3′ Mouse Il18 antisense 5′‐TGTTCTTACAGGAGAGGGTAGAC‐3′; Mouse Tnf sense 5′‐ GGTGCCTATGTCTCAGCCTCTT‐3′ Mouse Tnf antisense 5′‐GCCATAGAAC TGATGAGAGGGAG‐3′; Mouse Il6 sense 5′‐TACCACTTCACAAGTCGGAGGC‐3′ Mouse Il6 antisense 5′‐CTGCAAGTGCATCATCGTTGTTC‐3′;

### 2.7 ELISA

The IL‐1β, IL‐18, and TNF‐α levels in serum, cell culture supernatant, and gingival tissues were measured using corresponding commercial ELISA kits following the manufacturer's instructions (Abcam, China).

### 2.8 Immunoprecipitation and Western blot

The immunoprecipitation and western blot were performed following standard protocols as described previously.^39^ Plasmid‐encoding Myc‐Synoviolin and plasmid‐encoding Flag‐GSDMD were co‐transfected into HEK293 T cells using Lipofectamine 2000 (Thermo Fisher). Cells were lysed in RIPA buffer (Abcam, China), and the supernatant was subjected to immunoprecipitation using anti‐Flag antibody. Then, the samples were subjected to SDS‐PAGE and transfer. Antibodies used in this study included: Antibody for Actin (C‐4, 1:10000) was purchased from Sigma; GSDMD (E9S1X) Rabbit mAb (39754), Caspase‐1 Antibody (2225), Synoviolin (D3O2A), Rabbit mAb (14773), and Ubiquitin (P4D1) Mouse mAb (3936) were purchased from Cell Signaling Technology.

### 2.9 Statistical analysis

The results were shown as mean ± SD. Statistical analysis was performed using SPSS software (SPSS). ANOVA analysis with Scheffe's Test was used for statistics. Jarque–Bera and D'Agostino tests were used as normality tests. The significance level was set at *p* < .05. The bar graphs were drawn by GraphPad Prism software (GraphPad Software).

## 3 RESULTS

### 3.1 Activation of inflammasome was associated with periodontitis

**Figure 1:**
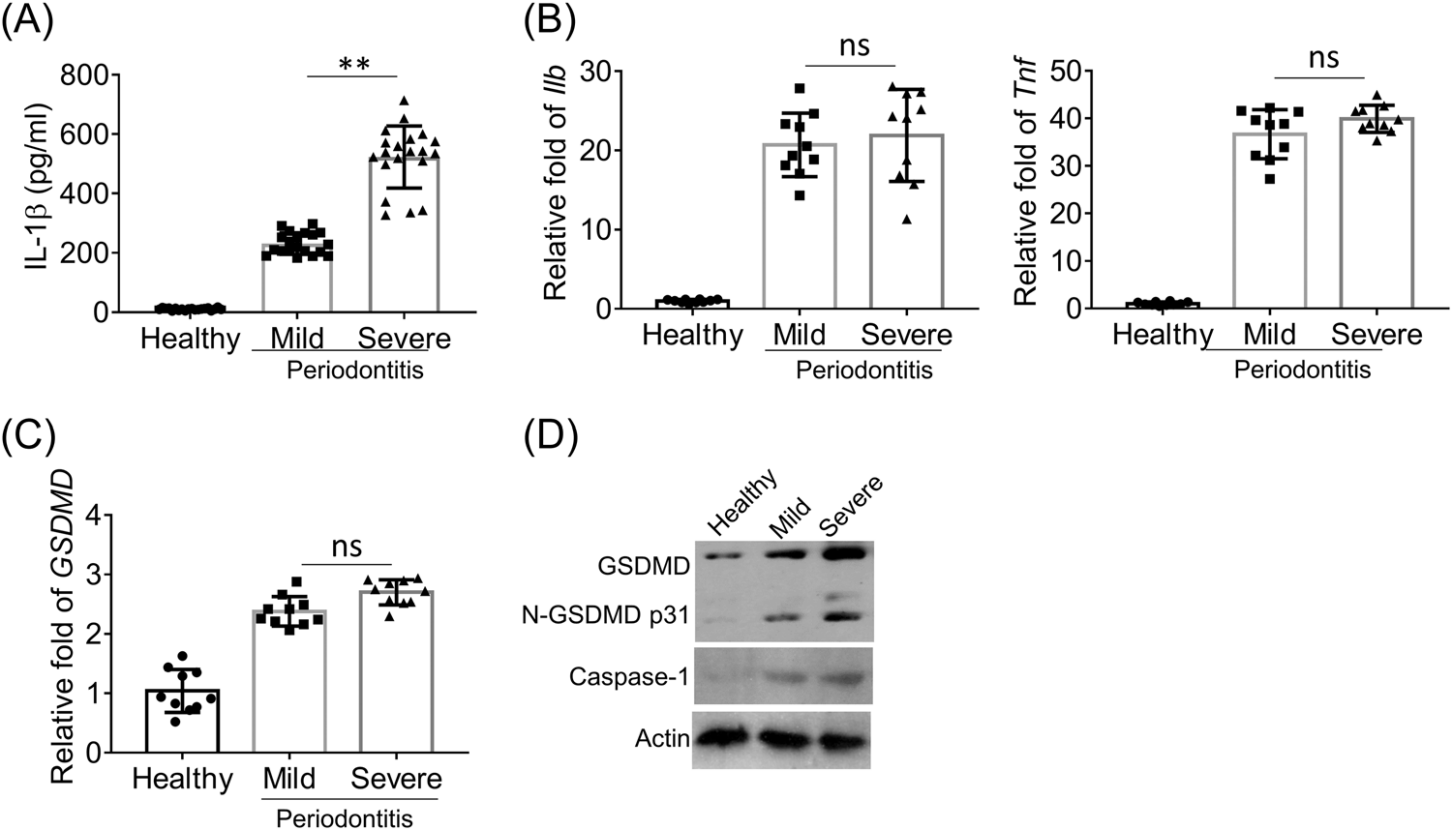
The activity of inflammasome is involved in the onset of periodontitis. (A) Enzyme‐linked immunosorbent assay (ELISA) of the IL‐1β in the serum of healthy donors and patients with mild and severe periodontitis (*n* = 20 each group). (B and C) The mRNA levels of Il1β and Tnf (B) and gasdermin D (GSDMD) (C) in peripheral blood mononuclear cells (PBMCs) of healthy donors and the patients with mild and severe periodontitis were assessed by quantitative real‐time polymerase chain reaction (RT‐PCR) (*n* = 9). (D) The protein levels of GSDMD and Caspase‐1 in PBMCs of healthy donors and patients with mild and severe periodontitis were measured by Western blot. Data are presented as mean ± SD values and are representative of at least three independent experiments. **p < .01.

The serum level of IL‐1β in healthy donors, patients with mild periodontitis, and patients with severe periodontitis was determined by ELISA. As presented in Figure 1A, minimal IL‐1β was detected in the serum of healthy donors. In contrast, remarkably elevated IL‐1β level was detected in the serum of patients with mild and severe periodontitis. Furthermore, the IL‐1β level in the serum of patients with severe periodontitis was significantly higher than in the serum of patients with mild periodontitis. We detected significantly increased mRNA levels of IL‐1β and TNF‐α in PBMCs isolated from patients with mild and severe periodontitis, while there was no difference in IL‐1β and TNF‐α mRNA levels between PBMCs isolated from patients with mild periodontitis and PBMCs isolated from patients with severe periodontitis (Figure 1B). We also detected remarkably elevated mRNA levels of GSDMD in PBMCs isolated from patients with mild periodontitis and PBMCs isolated from patients with severe periodontitis, while there was no difference between mild periodontitis and severe periodontitis (Figure 1C). Correspondingly, we detected obviously increased protein amounts of cleaved GSDMD (p31) and caspase‐1 (p20) in PBMCs isolated from patients with mild and severe periodontitis (Figure 1D). Interestingly, the protein level of GSDMD and p31 in PBMC isolated from patients with severe periodontitis was higher than in PBMCs isolated from patients with mild periodontitis. Taken together, these results demonstrated that in periodontitis, the inflammasome was activated.

### 3.2 Synoviolin interacted with GSDMD and regulated its ubiquitination

**Figure 2:**
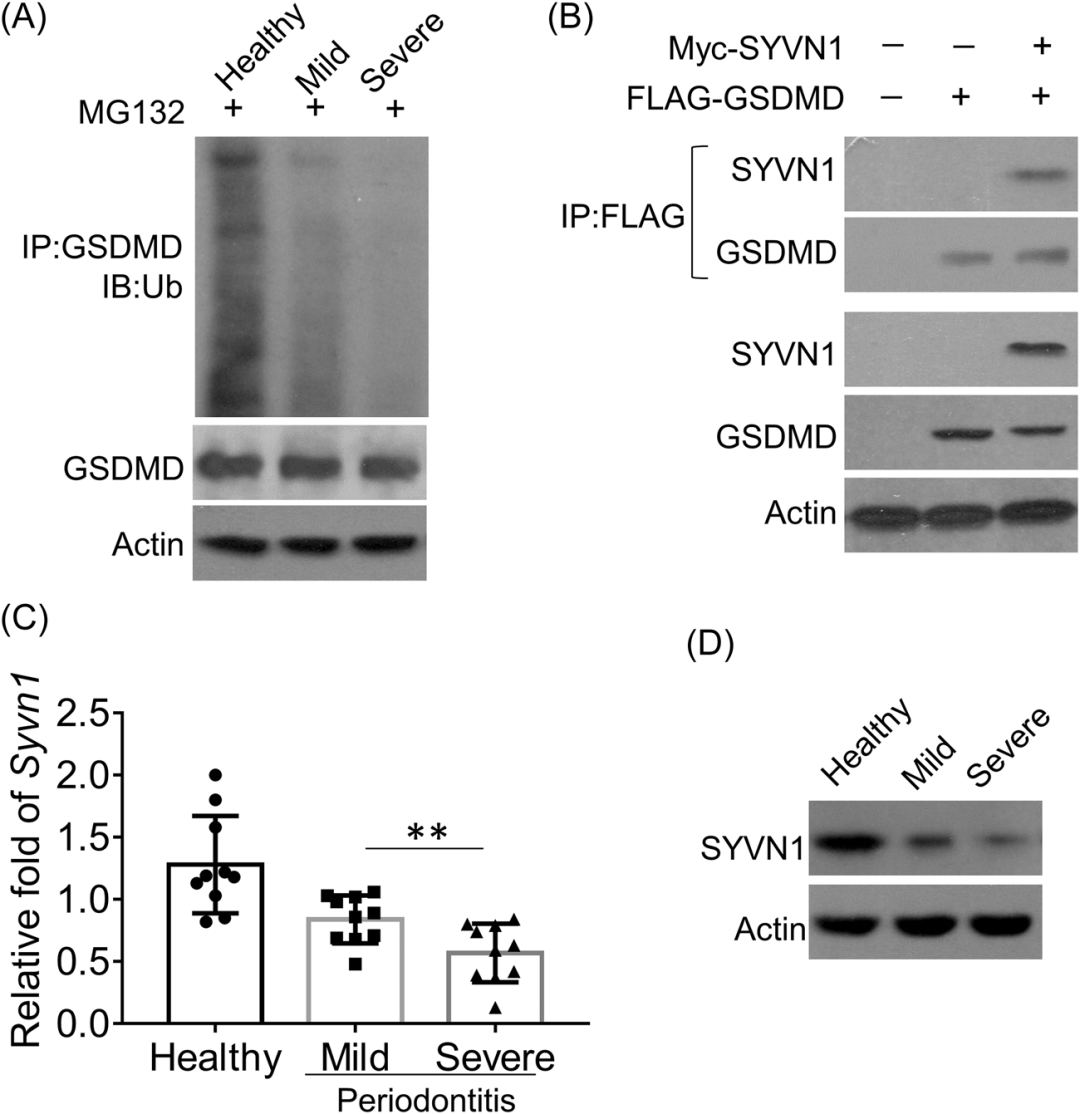
Synoviolin (SYVN1) regulated the protein stability and ubiquitination of gasdermin D (GSDMD). (A) GSDMD was isolated by Immunoprecipitation from whole‐cell lysates of peripheral blood mononuclear cells (PBMCs) of healthy donors and patients with mild and severe periodontitis and subjected to Western blot (IB) assays using antiubiquitin. Protein lysates were also subjected to direct IB. (B) Immunoassay of HEK293T cells transfected with various combinations of expression vectors followed by immunoprecipitation of proteins from lysates with anti‐FLAG or control IgG and immunoblot analysis with Myc. (C) The mRNA level of SYVN1 in PBMCs of healthy donors and patients with mild and severe periodontitis was assessed by quantitative real‐time polymerase chain reaction (RT‐PCR) (*n* = 9). (D) The protein levels of SYVN1 in PBMCs of healthy donors and patients with mild and severe periodontitis were measured by IB. Data are presented as mean ± SD values and are representative of at least three independent experiments. **p < .01.

The stability of GSDMD was regulated by ubiquitination.^40^ In PBMCs isolated from patients with mild and severe periodontitis, the ubiquitination of GSDMD was obviously decreased compared to PBMCs isolated from healthy donors (Figure 2A). In addition, the ubiquitination of GSDMD in PBMCs isolated from patients with severe periodontitis was further decreased. The E3 ligase Synoviolin has been shown to interact with GSDMD.^36^ We co‐transfected plasmid‐encoding Flag‐tagged Synoviolin with plasmid‐encoding GSDMD to HEK293T cells and confirmed the interaction of Synoviolin and GSDMD by immunoprecipitation (Figure 2B). We further found that both mRNA and protein levels of Synoviolin were remarkably decreased in PBMCs isolated from patients with mild and severe periodontitis (Figure 2C,D).

### 3.3 Synoviolin suppressed the secretion of IL‐1β and IL‐18

**Figure 3:**
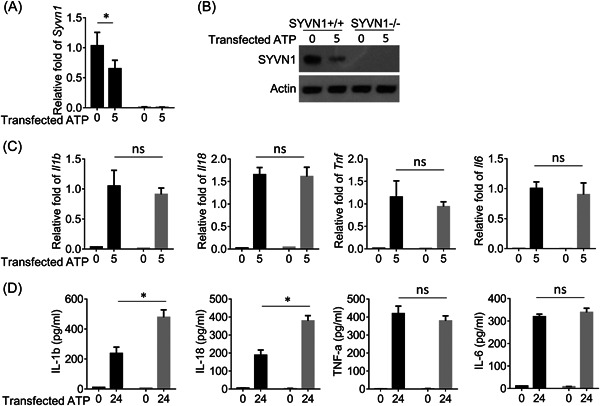
Synoviolin (SYVN1) suppresses the secretion of IL‐1β and IL‐18 by regulating inflammasome. (A and B) Quantitative real‐time polymerase chain reaction (qRT‐PCR) analysis and Western blot showing the expressions of SYVN1 in WT and SYVN1 KO bone marrow‐derived macrophages (BMDMs). (C) The expression of indicated cytokines in WT and SYVN1 KO BMDMs was stimulated with transfected ATP and measured by qRT‐PCR. Actin was used as a loading control and for relative normalization. (D) Enzyme‐linked immunosorbent assay (ELISA) of the indicated cytokines in the supernatants of BMDMs described in C. Data are presented as mean ± SD values and representative of at least three independent experiments. *p < .05.

Next, we evaluated the effects of Synoviolin on the expression and secretion of IL‐1β and IL‐18 by using Synv1 KO BMDMs. We transfected ATP into WT and Synoviolin KO BMDMs and monitored the expression of Synoviolin, IL‐1β, and IL‐18. As presented in Figure 3A, ATP treatment significantly decreased the mRNA level of Synoviolin in WT BMDMs, and there was no obvious Synoviolin mRNA detected in Synoviolin KO BMDMs. Correspondingly, ATP treatment reduced the Synoviolin protein amount in WT BMDMs, and no Synoviolin protein was detected in Synoviolin KO BMDMs (Figure 3B). We continued to determine the mRNA level of inflammatory cytokines, including IL‐1β, IL‐18, TNF‐α, and IL‐6 in WT and Synoviolin KO BMDMs after ATP treatment. As presented in Figure 3C, ATP treatment induced the mRNA expression of these four cytokines in BMDMs, and similar mRNA levels were detected between WT and Synoviolin KO BMDMs after ATP treatment, indicating that Synoviolin deficiency did not affect ATP‐induced expression of IL‐1β, IL‐18, TNF‐α, and IL‐6. Interestingly, we detected significantly increased amounts of IL‐1β and IL‐18 in the supernatant of ATP‐treated Synoviolin KO BMDMs, indicating enhanced secretion of IL‐1β and IL‐18 in Synoviolin KO BMDMs (Figure 3D). In contrast, similar amounts of TNF‐α and IL‐6 were detected in the cell culture supernatant of ATP‐treated WT and Synoviolin KO BMDMs. Collectively, these results demonstrated that Synoviolin deficiency promoted the secretion of IL‐1β and IL‐18.

### 3.4 Synoviolin deficiency promoted IL‐1β and IL‐18 secretion in mice with periodontitis

**Figure 4:**
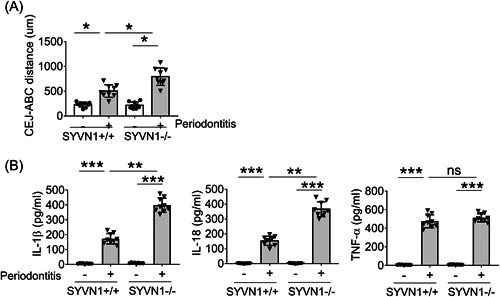
Synoviolin (SYVN1) deficiency promotes the activation of inflammasome in the periodontitis model. (A) The SYVN1fl/fl‐lyz2cre/+ (−/−) mice in the periodontitis model were treated as indicated. The cemento‐enamel‐junction–alveolar bone crest was analyzed by micro‐CT and plotted (*n* = 10 for each group). Averages and SD are shown. (B) mRNA levels in gingival tissues from these mice with periodontitis were examined by quantitative real‐time polymerase chain reaction (qRT‐PCR). All data are presented as fold relative to the Actb mRNA level. Data are presented as mean ± SD values and are representative of at least three independent experiments. *p < .05, **p < .01 and ***p < .005.

Finally, we compared the periodontitis phenotypes between WT mice and Synoviolin conditional KO (Synoviolin deficiency in myeloid cells) mice. As shown in Figure 4A and Supporting Information: Figure S1, compared to normal mice, WT mice with periodontitis and Synoviolin conditional KO mice with periodontitis had significantly elevated CEJ‐ABC distance. In addition, the CEJ‐ABC distance in Synoviolin conditional KO mice with periodontitis was more than that in WT mice with periodontitis. We further compared the cytokine levels in gingival tissues. In mice with periodontitis, significantly increased level of IL‐1β, IL‐18, and TNF‐α was detected in gingival tissues (Figure 4B). In addition, Synoviolin conditional KO mice with periodontitis had remarkably higher levels of IL‐1β and IL‐18 than WT mice with periodontitis in gingival tissues. In contrast, there was no difference in TNF‐α level in gingival tissues between WT mice and Synoviolin conditional KO mice with periodontitis.

## 4 DISCUSSION

Pyroptosis is a process of cellular self‐destruction which is involved in periodontitis. GSDMD, the key factor in pyroptosis, is regulated by ubiquitination.^40^, ^41^ Here, we evaluated the effects of the E3 ligase Synoviolin on GSDMD expression and on periodontitis. We detected upregulated expression and enhanced cleavage of GSDMD in PBMCs from patients with periodontitis, which was positively associated with the severity of periodontitis in patients. We confirmed the interaction between Synoviolin and GSDMD by immunoprecipitation in vitro and found that the expression of Synoviolin was decreased in PBMCs isolated from patients with periodontitis, which was correlated to decreased ubiquitination of GSDMD. We further demonstrated that the deficiency of Synoviolin did not affect the expression of proinflammatory cytokines while affecting the secretion of IL‐1β and IL‐18 in ATP‐treated BMDMs. Finally, we found that mice with Synoviolin deficiency in myeloid cells had more severe periodontitis and enhanced secretion of IL‐1β and IL‐18 in gingival tissues. Therefore, our data from in vitro and in vivo experiments elucidated that Synoviolin mediated the ubiquitination of GSDMD, which regulated the stability of GSDMD and the activation of inflammasome in periodontitis.

Pyroptosis is an inflammatory programmed cell death that depends on caspases and GSDMD. The activation of inflammasome and caspase‐1 results in the cleavage of GSDMD, pro‐IL‐1β, and pro‐IL‐18. Mature GSDMD (N terminal fragment of GSDMD) forms pore‐structure in the plasma membrane, which enables the secretion of cellular components and leads to pyroptosis. Clinical studies revealed that pyroptosis was positively correlated with the severity of periodontal diseases.^42^ In the present study, we found that there was upregulated mRNA expression of IL‐1β and GSDMD in PBMCs, as well as an increased serum level of IL‐1β in patients with periodontitis, suggesting the activation of inflammasome in periodontitis. Although there was no difference in the mRNA level of IL‐1β and GSDMD between patients with mild periodontitis and patients with severe periodontitis, significantly elevated IL‐1β protein in the serum of patients with severe periodontitis was detected. The protein level of GSDMD in PBMCs from patients with severe periodontitis was higher than that in PBMCs from patients with mild periodontitis. These results strongly suggested that the activation of inflammasome and pyroptosis was positively correlated with the severity of periodontitis, which was regulated at the protein level but not the mRNA level. It has been described that ubiquitination regulated GSDMD. In the study by Zhu et al., the authors demonstrated that ubiquitination regulated GSDMD and affected its degradation.^40^ They observed that sodium arsenite (NaAsO₂) reduced the K48‐ and K63‐linked ubiquitination of GSDMD and prevented its degradation through the ubiquitin‐proteasome system and the autophagy‐lysosome pathway, which promoted pyroptosis. In the present study, we also observed the ubiquitination of GSDMD in PBMCs from healthy donors. In contrast, the ubiquitination of GSDMD was obviously decreased in PBMCs from patients with periodontitis, which indicated the suppression of GSDMD ubiquitination in these PBMCs. The suppression of GSDMD ubiquitination was correlated to the increased protein level of GSDMD, which would contribute to the promoted activation of inflammasome and secretion of IL‐1β and IL‐18. Until now, the E3 ligases involved in the ubiquitination of GSDMD are not fully elucidated yet. The study by Shi and colleagues described that E3 ligase Synoviolin directly interacted with GSDMD and mediated K27‐linked polyubiquitination of GSDMD, which promoted GSDMD‐induced pyroptosis.^36^ In the present study, we also confirmed the interaction between Synoviolin and GSDMD. Interestingly, we found the downregulation of Synoviolin in periodontitis, which was correlated to the decreased ubiquitination of GSDMD, suggesting Synoviolin was responsible for GSDMD ubiquitination in periodontitis. The decreased ubiquitination of GSDMD may result in enhanced stability of GSDMD. Unfortunately, in the present study, we did not distinguish the specific lysine linkage of GSDMD in periodontitis. Previous studies revealed that the K27‐linked polyubiquitination of GSDMD was involved in its activity, while K48‐ and K63‐linked ubiquitination of GSDMD was involved in stability and degradation. It should be useful to further characterize lysine linkage of GSDMD in PBMCs from patients with periodontitis. In the present study, we generated Synoviolin conditional KO mice and found that these mice produced more mature IL‐1β and IL‐18 and had exacerbated periodontitis, suggesting that Synoviolin played a protective role in periodontitis by suppressing inflammasome activation. To better understand the roles of Synoviolin in periodontitis, Synoviolin knock‐in mice may also be considered. It should be reasonable to speculate that mice overexpressing Synoviolin in myeloid cells could have promoted GSDMD degradation and suppressed inflammation. Besides GSDMD, Synoviolin also targeted other proteins. It is also possible that Synoviolin protects against periodontitis by regulating other factors. Experiments need to be designed to explore the other possibilities.

In this study, we utilized a Pg‐induced periodontitis mice model. Another murine model of periodontitis, such as ligation, has been widely used to simulate alveolar bone resorption and periodontal soft tissue destruction.^43^ Therefore, it is worth testing the effects of Synoviolin on periodontitis using this model.

## 5 CONCLUSION

In the present study, we found that Synoviolin was downregulated in PBMCs from patients with periodontitis, which was correlated to decreased ubiquitination of GSDMD, elevated GSDMD protein level, and increased inflammasome activation in these PBMCs. Synoviolin‐deficient BMDMs had increased IL‐1β and IL‐18 secretion while having similar TNF‐α secretion compared to WT BMDMs. Synoviolin deficiency in myeloid cells resulted in enhanced activation of the inflammasome and exacerbated periodontitis in mice with periodontitis. All these results demonstrated that Synoviolin protected against periodontitis by regulating GSDMD.

## AUTHOR CONTRIBUTIONS

**Ying Pang**: Conceptualization; data curation; writing—original draft; writing—review and editing. **Lili Liu**: Data curation; writing—original draft. **Shuainan Wu**: Data curation; writing—review and editing. **Jianqi Wang**: Data curation; writing—review and editing. **Lu Liu**: Data curation; writing—original draft; writing—review and editing.

## CONFLICT OF INTEREST STATEMENT

The authors declare no conflict of interest.

## ETHICS STATEMENT

All animal works were approved by the Institutional Animal Care and Use Committee in Cangzhou Central Hospital. This study was performed in strict accordance with the NIH guidelines for the care and use of laboratory animals (8th edition, NIH).

## Supporting information

Supporting information.

## Data Availability

Data would be made available upon request to the corresponding author.
