# Supplementary material for: Synoviolin alleviates GSDMD‐mediated periodontitis by suppressing its stability
Source: Immun Inflamm Dis. 2023 Jul 12;11(7):e880. doi: 10.1002/iid3.880 (PMC10336677; doi:10.1002/iid3.880)
Supplement: Supplementary file 1 — Supporting information. [file IID3-11-e880-s001.docx]

**Supplementary materials**


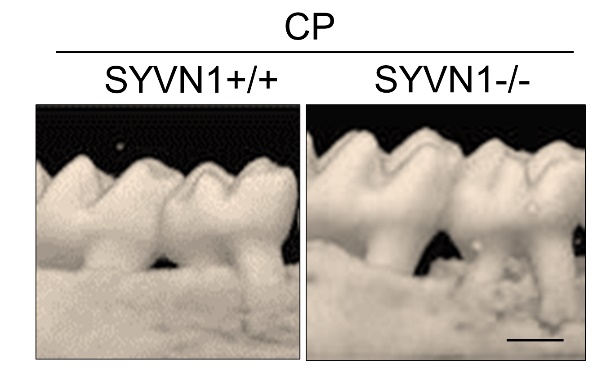


Figure S1. The cemento-enamel-junction–alveolar bone crest was analyzed by micro-CT. Scale bar=500 μm.
